# Mucosal T-cell responses to chronic viral infections: Implications for vaccine design

**DOI:** 10.1038/s41423-024-01140-2

**Published:** 2024-03-08

**Authors:** Mohammed Al-Talib, Sandra Dimonte, Ian R. Humphreys

**Affiliations:** 1 Systems Immunity University Research Institute/Division of Infection and Immunity, School of Medicine, Cardiff University, Cardiff, CF14 4XN UK; 2 Bristol Medical School, University of Bristol, 5 Tyndall Avenue, Bristol, BS8 1UD UK

**Keywords:** Virus, T cells, Mucosa, Chronic infection, Cytomegalovirus, HIV, Mucosal immunology, Vaccines, Viral infection

## Abstract

Mucosal surfaces that line the respiratory, gastrointestinal and genitourinary tracts are the major interfaces between the immune system and the environment. Their unique immunological landscape is characterized by the necessity of balancing tolerance to commensal microorganisms and other innocuous exposures against protection from pathogenic threats such as viruses. Numerous pathogenic viruses, including herpesviruses and retroviruses, exploit this environment to establish chronic infection. Effector and regulatory T-cell populations, including effector and resident memory T cells, play instrumental roles in mediating the transition from acute to chronic infection, where a degree of viral replication is tolerated to minimize immunopathology. Persistent antigen exposure during chronic viral infection leads to the evolution and divergence of these responses. In this review, we discuss advances in the understanding of mucosal T-cell immunity during chronic viral infections and how features of T-cell responses develop in different chronic viral infections of the mucosa. We consider how insights into T-cell immunity at mucosal surfaces could inform vaccine strategies: not only to protect hosts from chronic viral infections but also to exploit viruses that can persist within mucosal surfaces as vaccine vectors.

## Introduction

The mucosal immune system can be considered in terms of its topography. Secreted factors, which include mucus, constitute a physical barrier to pathogen entry, and antimicrobial mediators such as lysozyme, lactoferrin, complement and secretory IgA make up an initial layer of defense. Mucosal epithelial cells contribute to immunity not only through their barrier function but also, as found more recently, through their ability to sense and regulate immune responses to viral infections [1]. Interspersed across the mucosal epithelial surface, specialized microfold (M) cells sample antigens from the mucosal surface into mucosal lymphoid tissues, such as Peyer’s patches in the small intestine. A repertoire of innate, innate-like and adaptive immune cells reside at mucosal surfaces and orchestrate and regulate the antiviral immune response [2]. Among these, diverse lineages of T cells, including tissue-resident memory T cells (T_RM_), effector memory T cells (T_EM_), regulatory T cells (T_REG_), and unconventional T cells (including mucosal-associated invariant T cells, natural killers and γδ T cells), play important roles in mediating a nuanced immune response as virus chronicity is established, where unrestrained inflammatory responses in the face of persistent antigen exposure would result in immunopathology and host tissue damage.

Herein, we review the roles that T-cell subsets play in chronic viral infections of mucosal tissues. We discuss recent advances in the understanding of the role of key T-cell subsets in these processes and consider the implications of this knowledge for the design of virus-targeting vaccines. We also consider the potential features of T-cell responses elicited by certain chronic virus infections that may be exploited for the induction of effective long-lived mucosal immunity by virus-based vaccination strategies.

## Pathogenesis of chronic viral infections of the mucosa

**Fig. 1:**
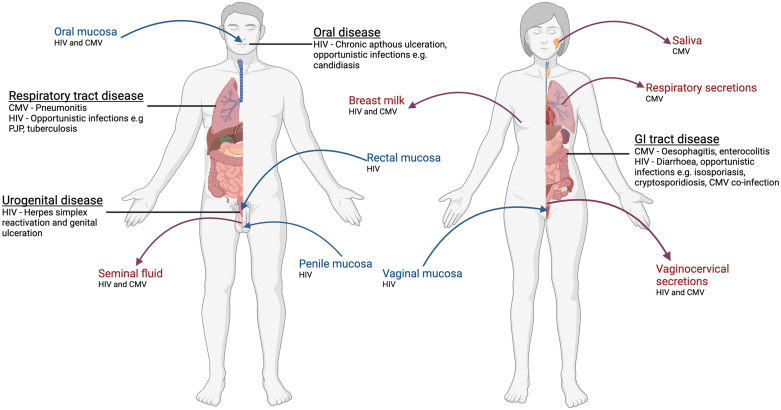
Major mucosal sites of acquisition, disease and transmission of CMV and HIV. Organs or tissues associated with infection are labeled blue for virus acquisition, red for viral transmission and black for virus-associated disease

Mucosal immunity is critical for pathogenesis and immune responsiveness to viruses. Mucosal surfaces are often sites of initial infection and can be major sites of persistence and/or latency [3, 4]. Furthermore, numerous chronic viral infections mediate their pathology within mucosal tissues, and some, such as Epstein–Barr virus (EBV) and human papilloma virus (HPV), can induce tumor formation in the mucosa [5, 6]. We first compare and contrast the roles that mucosal T-cell responses play in the pathogenesis and control of two important viral infections of the mucosa (Fig. 1), human cytomegalovirus (HCMV) and human immunodeficiency virus (HIV), and discuss features of these responses that are relevant to other chronic viral infections of the mucosa.

## Cytomegalovirus

Human cytomegalovirus (HCMV) is a ubiquitous β-herpesvirus that establishes lifelong infection. Chronic HCMV infection is underpinned by the establishment of latency and is facilitated by the expression of a plethora of immune evasion proteins that circumvent host immunity. HCMV infection in healthy individuals is typically asymptomatic, although HCMV infection may increase the risk of cardiovascular [7] and neurodegenerative [8] diseases and exacerbate frailty [9].

In immunocompromised individuals with perturbed T-cell responses, HCMV infection represents a significant clinical challenge. HCMV coinfection of HIV-infected individuals causes retinitis and vision loss, which may occur even after treatment with antiretroviral therapy (ART) [10, 11]. Severe gastrointestinal disease is commonly reported in HCMV coinfection [12], and HCMV actively replicates in the mucosal epithelium in patients receiving ART, disrupting epithelial integrity [13]. Despite improvements in surveillance and treatment, HCMV infection remains a significant clinical challenge in solid-organ and bone marrow transplant recipients receiving immunosuppressive therapy, including T-cell-depleting agents. HCMV infection is common in solid-organ transplant (SOT) patients, particularly HCMV-seronegative recipients (R^-^) receiving organs from HCMV-seropositive donors (D^+^). HCMV syndrome typically consists of malaise, fever and leukopenia as well as mucosal-associated conditions, including pneumonitis, gastrointestinal disease and nephritis [12]. In stem cell transplantation (SCT), disease is common in HCMV-seropositive individuals, where the virus reactivates from latency. Mucosal-associated diseases, particularly pneumonitis and enterocolitis, are common manifestations [10].

HCMV is also a major cause of congenital infection, particularly in developing countries where incidence rates above 1% have been reported [14]. Congenital HCMV is the leading cause of non-hereditary sensorineural hearing loss (SNHL) and can cause severe and permanent neurological defects leading to complications including intellectual disability and seizures [15]. The cost of congenital HCMV in the UK alone was estimated in 2016 to be ~£750M [16]. The human and economic costs of congenital HCMV infection highlight the need for an HCMV vaccine. The majority of infections occur as a consequence of mucosal contact, and HCMV is detected in bodily fluids secreted from mucosal surfaces, including breast milk, semen, cervical fluid, urine and saliva [17]. Increased usage of daycare centers for young children has been implicated in increases in HCMV seroprevalence in women of childbearing age, suggesting that young children are a source of virus that causes congenital infection [18]. Thus, targeting this phase of the HCMV lifecycle will likely be critical for developing an efficacious vaccine, and understanding the cellular immune responses that participate in mucosal antiviral immunity will inform these efforts.

## Human immunodeficiency virus (HIV)

HIV is a lentivirus that is transmitted predominantly across mucosal surfaces during sexual intercourse, although HIV can also be acquired by bloodborne exposure and vertical transmission. The vast majority of infections worldwide are caused by HIV-1, as HIV-2 is mainly restricted to West Africa and is recognized to be less pathogenic [19]. Initial infection may precipitate a seroconversion illness characterized by nonspecific constitutional symptoms that in most individuals are self-limiting. However, the resultant chronic HIV-1 infection is characterized by persistent viral replication and an inexorable decrease in CD4^+^ T-cell counts that, over approximately 10 years, results in acquired immunodeficiency syndrome (AIDS) and susceptibility to opportunistic infection and malignancy.

Mucosal surfaces are pivotal in determining HIV progression. Early massive depletion of gut-associated lymphoid tissue (GALT) results in mucosal disruption and increased microbial translocation, driving chronic immune activation and dysfunction and, later, AIDS progression. In addition, AIDS-defining illnesses occur at mucosal sites as a direct consequence of impaired mucosal immunity. For example, *Pneumocystis jirovecii* pneumonia, an opportunistic fungal infection and major cause of mortality in AIDS patients, is characterized by impaired innate and adaptive immune responses in the lung mucosa [20]. Other significant mucosal opportunistic infections include oesophageal or respiratory tract candidiasis, HCMV-associated gastrointestinal disease, chronic mucosal ulceration due to herpes simplex reactivation and chronic intestinal isosporiasis. Although ART has dramatically altered the prognostic outlook, HIV remains a major global health burden: at the time of writing, the World Health Organization estimates that almost 40 million people are infected with HIV, with 630,000 people dying of HIV-related causes in 2022 [21].

T cells are the predominant cellular targets of HIV replication. In transmission, HIV typically utilizes C-C chemokine receptor type 5 (CCR5), which is highly expressed on mucosal CD4^+^ T cells, to gain entry into host cells. HIV penetrates the mucosal barrier to successfully infect CD4^+^ T cells through several mechanisms. First, via the ‘virological synapse’, HIV can be transferred between dendritic cells (DCs) exposed to HIV and CD4^+^ T cells [22]. Furthermore, DC-mediated infection of CD4^+^ T cells may occur via the release of HIV-containing exosomes [23] and viral transmission in *cis*, whereby de novo replication within infected DCs results in progeny viruses that infect CD4^+^ T cells [24]. Once the initial infection is established, CCR5^+^ memory CD4^+^ T cells are rapidly depleted in GALT [25, 26], and the virus disseminates systemically.

HIV progression centers on chronic activation of CD4^+^ and CD8^+^ T cells in response to microbial translocation across the gastrointestinal barrier. In their seminal work, Brenchley et al. used circulating lipopolysaccharide (LPS) as a marker of bacterial translocation and demonstrated that this level was significantly increased in rhesus macaques chronically infected with SIV, which was a consequence of increased intestinal permeability mediated by the massive depletion of GALT [27]. Notably, natural SIV infection in sooty mangabeys, which is nonpathogenic, induces minimal bacterial translocation and low immune activation. Supporting this finding, microarray and qPCR analyses of GALT cells from HIV-infected patients indicated rapid disruption of mucosal integrity associated with the downregulation of genes associated with metabolism; mucosal growth, maintenance and repair; and increased expression of immune activation- and inflammation-associated genes [28]. This chronic immune activation ultimately leads to the progressive loss of CD4^+^ T cells. Indeed, the expression of CD38, a marker of CD8^+^ T-cell activation, is more strongly predictive of HIV progression than is the CD4 count or viral load [29].

Mucosal surfaces are also critical sites of HIV secretion and dissemination. Semen is the most important vector for HIV-1 transmission, with the viral load in seminal fluid mirroring the blood with slightly delayed dynamics [30]. In the acute stage, HIV in semen is genetically similar to the bloodborne virus, suggesting viral diffusion into the seminal lumen [31]. Semen also contains lymphocytes and germ cells, and transmitted HIV virions may be cell-free or cell-associated, and there is evidence that both can cause productive infection [32]. Other secreted sources of HIV that may facilitate transmission include cervical and vaginal secretions [33], saliva and breast milk [34].

## T-cell responses in the mucosa during chronic viral infections

Virus-specific T cells within mucosal tissues play influential and interconnected roles in mediating the host response against chronic viral infections, and these responses may promote viral control as well as immunopathology [35, 36]. Below, we highlight some of the major T-cell subsets that contribute to this nuanced response, focusing on HIV and CMV infections.

## αβ CD4^+^ effector T cells

### CD4^+^ T cells and CMV – key antiviral effector cells in mucosal tissues

**Fig. 2:**
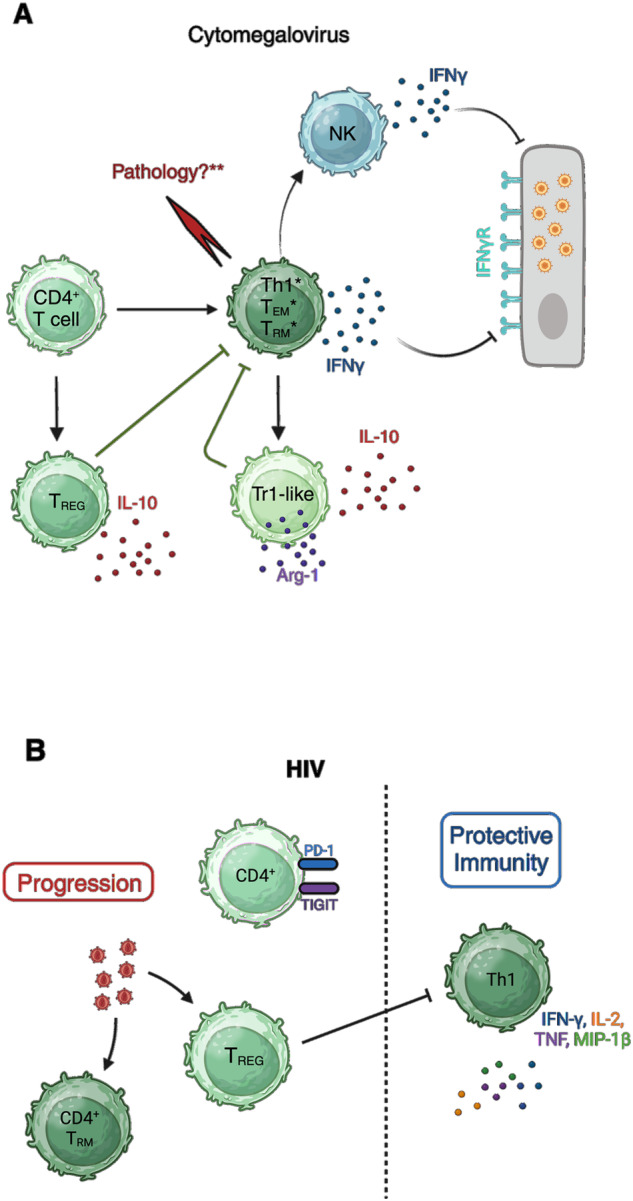
The role of CD4^+^ T-cell subsets in mucosal CMV and HIV infection. **A** Schematic representation of the role of virus-specific CD4^+^ T cells in mucosal CMV infection. *The exact contributions of effector Th1 cells, T_EM_ cells and T_RM_ cells to antiviral effector functions in different contexts and in the development of Tr1-like cells are not fully defined. **MCMV-induced CD4^+^ T cells mediate pathology if inadequately regulated, although it remains to be determined whether virus-specific cells drive tissue pathology. **B** Limited information regarding mucosal CD4^+^ T cells in HIV infection suggests preferential susceptibility of T_REG_ and T_RM_ cells to HIV infection. Data derived from elite controllers suggest that polyfunctional mucosal CD4^+^ T cells are correlated with protection. Created with BioRender.com

The importance of CD4^+^ T cells in antiviral control of CMV infection in mucosal tissues (Fig. 2A) is clearly demonstrated in the murine CMV (MCMV) model. Following systemic infection, MCMV replicates in multiple tissues, including the spleen, liver, kidneys and lung, prior to establishing persistence in the salivary glands (SGs) [37], where MCMV elicits robust CD4^+^ and CD8^+^ T-cell responses [38]. Interestingly, however, Jonjić and colleagues demonstrated that depletion of CD4^+^ but not CD8^+^ T cells dramatically increased the viral load at this site of virus persistence [39]. In the same experiments, the authors also demonstrated an antiviral role for CD4^+^ T cells in the respiratory mucosa [39], and adoptive transfer of transgenic CD4^+^ T cells specific for the viral M25-derived epitope limited MCMV replication in the SGs and lungs of immunocompromised hosts [40]. An antiviral role for CD4^+^ T cells in both the respiratory and oral mucosa is also supported by the observation that MCMV-mediated degradation of MHC class II by the viral M78 protein facilitates MCMV colonization of SGs following intranasal challenge [41].

MCMV-specific CD4^+^ T-cell responses exhibit broad specificity [42, 43]. Arens et al. demonstrated diverse cytokine expression profiles, including of IL-17 and the regulatory cytokine IL-10. However, early adoptive transfer studies suggest a role for IFNγ (but not TNF-α) in antiviral control [44]. Moreover, expression of IFNγ and/or TNF-α is a key feature of virus-specific CD4^+^ T cells that respond to an epitope derived from the viral m09 protein [43]. m09-specific CD4^+^ T cells are detectable only at later time points during MCMV infection, which coincides with the control of virus replication in SGs, suggesting a role for m09-specific Th1-like cells in the control of mucosal MCMV replication. In contrast, although the expression of granzyme B by MCMV-specific CD4^+^ T cells has been described [45], perforin is dispensable for the control of MCMV infection of SGs [40]. Instead, adoptive transfer studies suggest that, during MCMV infection of the lungs and SGs, CD4^+^ T-cell-derived IFNγ exerts direct antiviral control on virus-infected cells [40], although more recently, NK cells have also been implicated in mediating the antiviral functionality of CD4^+^ T cells [46].

Despite a defined role for CD4^+^ T-cell responses in HCMV infection, particularly in the transplant setting (reviewed elsewhere [47]), less is known about how these cells respond to mucosal HCMV infection. Th1-like [48] and cytolytic [49] CD4^+^ T cells develop during HCMV infection, suggesting parallels with the MCMV model. Furthermore, HCMV-specific CD4^+^ T cells in the periphery exhibit a phenotype indicative of differentiated memory cells (CD27^lo^CD28^lo^CD57^+^CD45RA^+^ [47]), and as observed in MCMV infection, HCMV-specific CD4^+^ T cells also react to a broad array of antigens [50]. Critically, the importance of CD4^+^ T cells in controlling mucosal HCMV infection has been demonstrated in immunocompetent young children. Tu et al. determined that the majority of circulating HCMV-specific IFNγ-expressing cells in adults and children were CCR7^low^, suggesting that these cells were T_EM_-like [51]. Strikingly, compared to adults, children had dramatically reduced Th1 responses and associated CCR7^low^ CD4^+^ T-cell populations, which was associated with prolonged viral shedding in the urine. In immunosuppressed individuals, HSCT patients provide the most compelling evidence for a role for mucosal CD4^+^ T cells in antiviral protection. CMV exposure induces long-term accumulation of virus-specific memory CD4^+^ T cells [52], and chronic CD4^+^ T-cell lymphopenia is correlated with the occurrence of CMV disease manifestations, including pneumonitis and gastroenteritis [53]. Reconstitution of CD4^+^ T cells aids in the control of HCMV reactivation and gastrointestinal disease in these patients [54]. Thus, data from HCMV patients imply the broad antiviral function of virus-specific CD4^+^ T cells in multiple mucosal tissues. Importantly, in the context of congenital CMV infection, data from the rhesus macaque model demonstrated that CD4^+^ T cells also limit transplacental transmission of CMV [55]. Overall, these data demonstrate that consideration of CD4^+^ T cells will be critical for developing efficacious HCMV vaccines to limit virus transmission and pathogenesis.

### CD4^+^ T cells and HIV - Targets of viral infection and antiviral effectors

Less direct evidence exists regarding the exact role of CD4^+^ T cells in the mucosa in HIV infection (Fig. 2B). However, in comparison to HCMV, their role differs considerably as virus chronicity is enabled by the establishment of reservoirs within CD4^+^ T cells, which facilitate persistent low-level viral replication and harbor latent virus during ART treatment. Although lymphoid tissues are a major site of HIV replication, evidence of proviral DNA in cells has been obtained from the lung [56], ileum [57] and cervix [58] of ART-treated patients. Viral DNA^+^ and RNA^+^ cells are also detectable across all tissue types in ART-treated macaques infected with SIV; however, the gastrointestinal tract harbors the largest viral reservoir [59].

Key insights into the contribution of virus-specific CD4^+^ T cells to antiviral responses in HIV infection have been derived by comparisons of immune responses between typical HIV-infected individuals and the small proportion who spontaneously and durably suppress viral replication and who do not progress to AIDS (so-called ‘elite controllers’). While much of this work has focused on peripheral T-cell responses [60, 61], there is evidence that virus-specific CD4^+^ T cells in the mucosa contribute to protection. Ferre et al demonstrated that HIV-specific CD4^+^ T cells isolated from the rectal mucosa of controllers exhibited higher IFN-γ, IL-2, TNF and MIP-1β responses than those isolated from ARTs and that this population was characterized by a higher frequency of polyfunctional CD4^+^ T cells [62]. Interestingly, the most pronounced mucosal polyfunctional CD4^+^ responses were seen in controllers harboring both HLA-DRB1*13 and HLA-DQB1*06. Similar HLA associations with viral control were identified in an earlier study, with higher-magnitude responses observed in individuals with the HLA-DRB1*13/HLA-DQB1*06 haplotype [63]. Furthermore, patients who fail to restore their CD4^+^ T-cell counts after ART treatment express the gut-homing marker ITGβ7 in peripheral CD4^+^ T cells at higher frequencies than patients who do recover their CD4^+^ T cell counts, and CD4^+^ T cells in the gastrointestinal mucosa of these individuals display a more exhausted (PD-1^+^TIGIT^+^) phenotype [64], suggesting the importance of functional CD4^+^ T cells in protecting the mucosa from HIV replication.

## αβ CD8^+^ effector T cells

### Protective and immunopathological ‘inflationary’ CD8^+^ T-cell responses in CMV infection

The effector memory CD8^+^ T-cell responses elicited by CMV are remarkable. The ability of CMV to induce high frequencies of virus-specific memory CD8^+^ T cells was first demonstrated in the lungs of MCMV-infected BALB/c mice following systemic infection [65], and the ability of MCMV to induce large systemic CD8^+^ T-cell responses over time was termed ‘memory inflation’ [66]. Subsequent studies revealed that this phenomenon also occurs in C57BL/6 mice [67, 68], suggesting that memory inflation is a broad feature of CMV-specific CD8^+^ T cells. Accordingly, high frequencies of HCMV-specific CD8^+^ T cells with a T_EM_ or T_EMRA_ phenotype in peripheral blood and mucosal tissues have been described [50, 69].

Inflationary CD8^+^ T-cell responses exhibit key features that differ from those of classical virus-specific T-cell responses (reviewed elsewhere [70, 71]). First, these cells do not contract following the acute phase of virus replication but increase to high frequencies [66–68] in a process that requires chronic antigenic stimulation [68] and help from CD4^+^ T cells [72–74]. Second, these cells exhibited a classical T_EM_ phenotype characterized by high CD44 expression and concurrent low expression of markers such as CCR7, CD62L, CD28, CD27 and CD127 [68, 75], suggesting that these cells are distributed in the periphery rather than in secondary lymphoid tissue. Interestingly, unlike in some other chronic virus infections, CMV-specific CD8^+^ T cells in the periphery express low levels of inhibitory receptors, such as PD-1 [76] (although CMV-specific T_RM_ express PD-1, discussed later). Accordingly, CMV-specific CD8^+^ T cells maintain their functionality over time, exhibit proliferative and cytotoxic effects and readily express antiviral cytokines [70, 71]. Although inflationary CD8^+^ T cells have been described predominantly in CMV infection, there is also evidence that other mucosal chronic viral infections, including norovirus [77] and, in certain contexts, EBV [78], can induce inflationary CD8^+^ T cells. As discussed later in this review, these responses can also be induced by adenovirus-based vectors, further demonstrating that this phenomenon is not restricted to CMV.

As discussed, CD8^+^ T cells do not restrict primary MCMV infection in SGs [39, 44] although they accumulate at high frequencies [38, 44]. MCMV efficiently downregulates MHC class I during lytic infection, particularly in acinar glandular epithelial cells [79, 80]. However, memory CD8^+^ T cells (which may include T_RM_ cells) restrict MCMV reactivation from latency in the lungs and salivary glands [81] and, following adoptive transfer into immunocompromised hosts, limit MCMV replication in the lungs [82, 83]. Additionally, reconstitution of CD8^+^ T cells in adult and pediatric HSCT patients restricts CMV pneumonia [84, 85]. This approach is also efficacious in these patients against EBV and human adenovirus [86], which replicate and cause disease in tissues including mucosa. Another function of inflationary CD8^+^ T cells is to seed T_RM_ populations in mucosal tissues [87]. Thus, effector CD8^+^ T cells respond to CMV through important antiviral functions despite the use of viral immune evasion strategies to abrogate these responses.

Despite the antiviral functions of inflationary CD8^+^ T-cell responses in CMV infection, there are potential detrimental impacts of high-frequency T-cell responses to a single pathogen. HCMV is implicated as a driver of immunosenescence [88]. Furthermore, in both immunocompetent and immunocompromised individuals, HCMV causes disease in multiple tissues, including the mucosa, and, in many cases, T-cell influx at the site of pathology is associated with inflammation [35]. Similarly, conflicting roles for CD8^+^ T-cell responses in establishing viral control and mediating immunopathology have been suggested in BK polyomavirus (BKPyV) infection. BKPyV is a ubiquitous human polyomavirus that resides in the kidney and urinary tract and, in immunosuppressed kidney transplant recipients (KTRs), can drive pathology, including graft damage [89]. Viral control in KTRs is temporally correlated with the development of BK-specific IFNγ^+^ T-cell responses [90, 91]. However, immunopathology associated with BKPyV is characterized by tubulitis and interstitial lymphocytic infiltration, including by CD8^+^ T cells [92]. As with HCMV, it is unclear whether tissue damage is a bystander consequence of virus-specific T-cell responses or the result of viral replication and local tissue damage promoting the recruitment of T cells.

### Sustained HIV replication drives CD8^+^ T-cell exhaustion and disease progression

Nearly all HIV-infected individuals mount a virus-specific CD8^+^ T-cell response, and the importance of these T cells in antiviral protection is demonstrated in elite controllers [93]. Although much research has focused on peripheral CD8^+^ T cells [94–96], there is evidence of more robust mucosal HIV-specific CD8^+^ T-cell responses among controllers associated with the improved ability of CD8^+^ T cells to traffic to sites of HIV persistence, including the gastrointestinal mucosa [97]. Further, polyfunctional CD8^+^ T-cell responses (including IFNγ, IL-2, TNF and CD107a) among these controllers were enhanced, with stronger responses from cells derived from the rectal mucosa than from blood.

While effective virus-specific CD8^+^ T-cell responses play a role in protection against disease progression in controllers, an inadequate early response portends CD8^+^ T-cell dysfunction, which is a characteristic feature of progression to AIDS in most HIV-infected individuals. Using an SIV rhesus macaque model of vaginal transmission and early infection, Reynolds and colleagues showed that CD8^+^ T-cell responses to immunodominant SIV epitopes in the vaginal mucosa (and blood) develop after the peak of viraemia, at which time SIV had already disseminated throughout lymphoid tissues [98]. Interestingly, virus-specific responses in the GALT were the lowest of any compartment tested. Whether this phenomenon reflects an intrinsic viral mechanism that facilitates infection and depletion of GALT T cells is unclear. The observation that intravenous inoculation generates more robust GALT responses [99] highlights the importance of studying mucosal immunity to lentiviral, and chronic viral infections generally, in the context of relevant and specific mechanisms of transmission. Furthermore, CD8^+^ T cells isolated from the gastrointestinal mucosa have altered functionality, whereby cytolytic capacity is lower than that of peripheral T cells irrespective of HIV serostatus [100]. These observations are of particular relevance when considering the optimum delivery mode and potential efficacy of novel viral vaccine vectors and therapeutic approaches for HIV infection. Interestingly, pharmacological PD-1 blockade in SIV-infected rhesus macaques results in expansion and increased functionality of HIV-specific CD8^+^ T cells in the colorectal mucosa and blood [101]. Whether checkpoint inhibition represents a viable strategy for the treatment of HIV remains to be seen.

### Lessons from other chronic viral infections

Studies of other chronic viral infections also provide insights into features of the mucosal CD8^+^ T-cell response. Altered CD8^+^ T-cell functionality conditioned by the mucosal niche has been described in chronic norovirus (NoV) infection and can cause long-term and debilitating gastrointestinal symptoms in immunocompromised hosts. Inoculation of mice with a NoV strain that causes persistent infection results in a lower magnitude and functionality of intestinal mucosal virus-specific CD8^+^ T-cell responses compared to a NoV strain that is cleared acutely [102]. Furthermore, chronic murine NoV infection induces an effector memory phenotypic profile among intestinal NoV-specific CD8^+^ T cells, and although these cells retain functional responsiveness, they fail to respond when adoptively transferred into chronically infected mice, either due to a maladaptive functional profile or due to NoV utilizing an immune-privileged enteric niche to evade recognition [77]. The latter is suggested by work identifying murine NoV tropism toward intestinal tuft cells, which proliferate in response to type 2 cytokines, including IL-4, and, following exogenous IL-4 administration, promote enhanced viral replication and shedding [103]. In a mouse model of lethal herpes simplex virus-2 (HSV-2) infection, Arkatkar and colleagues recently found that the induction of antigen nonspecific memory CD8^+^ T cells by the administration of an irrelevant antigen delayed disease progression following HSV-2 challenge. This was a consequence of TCR-independent bystander activation of cells recruited into the inflammatory mucosal microenvironment [104]. These authors suggest that this principle may theoretically be harnessed in viral vaccine design and, while similar protection against bacterial infection has been observed in the lung mucosa [105], further characterization of this phenomenon in the context of other viral infections is needed.

## Mucosal tissue-resident memory T cells (T_RM_)

Over the past decade, there has been increasing recognition of the key role of T_RM_ cells, which are noncirculating memory T cells that reside in nonlymphoid tissues (NLTs), including the mucosa, in amplifying tissue-specific responses during reinfection that are more pronounced than the responses of circulating effector memory T cells (_EM_) [106–108]. T_RM_ across mucosal surfaces are defined by downregulation of genes associated with tissue egress and classically express CD69 and the integrin CD103, although the expression of these in different mucosal tissues varies [109]. Parabiosis studies have demonstrated that T_RM_ cells are the most abundant memory T-cell subset in nonlymphoid tissues (NLTs), including mucosal surfaces [110]. In addition to mediating local immunosurveillance and rapid protection against reinfection, T_RM_ cells also contribute to the pool of circulating effector memory T cells [111].

### T_RM_ - mucosal anti-herpesvirus effector cells

Analysis of human donor organs revealed the presence of HCMV-specific T_RM_ in the lungs and small intestines [112]. Mechanistic insight into the induction and function of CMV-specific T_RM_ in mucosal tissues has been mostly derived from the MCMV model. Systemic infection induces high frequencies of virus-specific CD4^+^ and CD8^+^ T_RM_ cells in SGs and small intestines [87, 113]. Moreover, intranasal MCMV challenge induces robust T_RM_ responses in nasal tissue [114] but, interestingly, not the lungs [115]. Following systemic MCMV infection, CD4^+^ T_RM_ formation is antigen-dependent, whereas CD8^+^ T_RM_ development is not [113]. SG T_RM_ are mostly derived from peripheral T-cell responses [113], and inflationary CD8^+^ T cells can migrate to the mucosa and form T_RM_, highlighting the dynamic nature of CMV-specific T-cell subsets [87]. HCMV- and MCMV-specific T_RM_ express PD-1, but these cells appear not to exhibit an exhausted phenotype [116, 117]. In accordance, CD8^+^ T_RM_ protect against intraglandular MCMV challenge [113]. The observation that T_RM_ but not effector CD8^+^ T cells control virus replication in SGs is attributed to their rapid responsiveness prior to viral downregulation of MHC class I [113]. Although the ability of Rhesus CMV (RhCMV) to infect hosts requires virus targeting of the MHC class I [118], it is possible that mucosal vaccination strategies to induce virus-specific T_RM_ on mucosal surfaces that respond rapidly to viral challenge could offer protection from horizontal CMV transmission.

Supporting the importance of T_RM_ cells in the control of mucosal herpesvirus infections, mice lacking vaginal CD4^+^ T_RM_ cells are unable to suppress HSV-2 replication following reinfection [119]. CD4^+^ and CD8^+^ T_RM_ cells in HSV-2-infected genital tissues are heterogeneously dispersed, and mathematical modeling suggests that at lower densities, T_RM_ cells initiate a polyfunctional cytokine response that activates bystander T cells and  controls replication [120]. Additionally, using a ‘prime and pull’ vaccination strategy against HSV-2 in mice elicited a vaginal IFNγ^+^ mucosal T_RM_ cell response, which abrogated clinical disease following viral challenge [121, 122].

### T_RM_ in HIV – antiviral effectors and viral reservoirs?

Interpreting the role of T_RM_ cells in HIV infection is challenging. Mucosal CD69^+^CD4^+^ T cells are major viral targets in acute infection and are rapidly depleted [123, 124], and CD4^+^ T_RM_ cells in the cervical mucosa may act as major cellular reservoirs of HIV [58]. Whether this is also the case in the GI tract, the major viral reservoir in ART-treated individuals [59] and a major site of CD4^+^ T_RM_ [116], is unclear. Conversely, CD8^+^ T_RM_ cells may control mucosal HIV infection. In the recto-sigmoid mucosal tract, most CD8^+^ T cells exhibit T_RM_ phenotypes and demonstrate polyfunctional characteristics that are strongest in elite controllers [125]. Of particular interest, a vaccination strategy consisting of three heterologous viral vectors that induced T_RM_ decreased the threshold of neutralizing antibodies required to provide durable resistance to simian-HIV challenge [126]. Additionally, T_RM_ activation in mucosal tissues triggers a local increase in vascular permeability that promotes exudation of virus-specific neutralizing antibodies [127]. Thus, T_RM_ may have broad antiviral functionality in HIV infection.

Overall, although potentially contributing to the HIV reservoir in ART-treated individuals, effective T_RM_ cell responses appear to be key contributors to protection against chronic viral infections of the mucosa. As discussed later, vaccine strategies promoting the induction of T_RM_ cells may offer robust protection against a range of mucosal pathogens.

## Inhibitory T cells

To limit infection-induced pathology during chronic infection of the mucosa, equilibrium is required where viral replication is tolerated without overzealous immune responses or associated tissue pathology. As highlighted by HIV infection, T-cell exhaustion is one mechanism through which pathological consequences of prolonged T-cell activation are abrogated. Another is the induction of regulatory T-cell populations.

### Diverse functions and phenotypes of suppressive CD4^+^ T cells in CMV infection

The potential role of inhibitory T cells in facilitating CMV chronicity in the mucosa was first suggested by Ann Campbell and colleagues, who described the expression of the immunoregulatory cytokine IL-10 in MCMV-infected SGs [38]. It was subsequently demonstrated that antagonizing IL-10R signaling, specifically during the chronic phase of infection, profoundly reduced virus replication [128], although this antiviral effect was not observed in the nasal mucosa [114]. SG FoxP3^-^IL-10^+^CD4^+^ T cells were defined in this initial study, and subsequently, MCMV-specific IL-10-secreting CD4^+^ T cells were defined [42, 43, 129], including those in SGs [130]. These cells lack classical Tr1 markers [130] but express T-Bet and EOMES and require T-Bet for their development, consistent with these cells being T-Bet-dependent Tr1 cells [131]. Importantly, conditional deletion of IL-10 in T cells improves control of MCMV persistence [130]. Thus, although IL-10 production during acute systemic MCMV infection inhibits CD4^+^ T-cell priming via suppression of NK-DC crosstalk [132], these data demonstrate that virus-specific IL-10^+^ CD4^+^ T cells develop in response to CMV infection and that IL-10 production by these cells profoundly inhibits T-cell immunity. IL-10-secreting CD4^+^ T cells also express arginase-1 (arg1) [131], which promotes the catalytic breakdown of L-arginine, an amino acid required for T-cell proliferation [133, 134]. T-cell-expressed arg1 also facilitates MCMV persistence [131], suggesting multiple immunoregulatory functions of these T cells.

During chronic MCMV infection of SGs, TRAIL-expressing NK cells restrict CD4^+^ T-cell numbers, and as a result of this limit a virus-induced autoimmune Sjogren’s-like syndrome [135]. Although it is unclear whether virus-specific CD4^+^ T cells mediate this process, these data clearly demonstrate that CD4^+^ T-cell responses triggered by chronic MCMV infection can drive autoimmunity. Importantly, however, deletion of IL-10 expression by CD4^+^ T cells during MCMV chronicity does not elicit the development of autoimmunity [130], suggesting that inhibiting the development of these cells could facilitate mucosal control of the virus without promoting tissue pathology. What is the origin of these cells, and can their development be abrogated? Recently, we used IL-10-reporter mice to study SG CD4^+^IL-10^+^ cells isolated during MCMV infection progression. Although MCMV-specific CD4^+^ T cells do not coexpress IL-10 or IFNγ in response to peptide stimulation [130], CD4^+^IL-10^+^ cells exhibit a gene signature indicative of Th1 cells [131] and coexpress the Th1-associated chemokine receptors CCR5 and CXCR3 [130]. IL-10^+^ CD4^+^ T cells lack clonal diversity and share clonotypes with IL-10^−^ CD4^+^ T cells, and their development is dependent upon the Th1-associated transcription factor T-Bet. Overall, these findings suggest that these cells represent clonally expanded Th1 cells that acquire IL-10 expression over time [131].

HCMV-specific IL-10^+^ CD4^+^ T cells have also been described, particularly cells reactive to latency-associated viral antigens [136]. The development of IL-10^+^ CD4^+^ T cells reactive to latency-associated proteins is also a hallmark of EBV infection [137], suggesting the development of IL-10-secreting T cells is a common feature of herpesvirus chronicity. Of note, in HCMV (and HIV) infection, IL-10-expressing CD8^+^ T cells have also been reported [138, 139], although the mechanisms that regulate CD8^+^ IL-10^+^ T-cell development and how these cells participate in mucosal infections are incompletely understood. T cells in the human colon express IL-10 in response to HCMV-derived gB and IE1 peptide pools [130]. Although these studies used ‘healthy’ tissue from cancer patients, these data suggest that HCMV chronicity leads to the development of IL-10^+^ T cells in human mucosal tissues.

Unlike MCMV, where the large majority of IL-10^+^ CD4^+^ T cells are FoxP3^-^, CD4^+^ T-cell cultures reactive to HCMV latency-associated antigens express FoxP3 [136]. Moreover, virus-induced T_REG_ cells suppress CMV-specific T-cell responses ex vivo [140], and inducible regulatory T-cell (iT_REG_) expansion is associated with reduced vascular pathology in elderly HCMV-infected individuals [141]. In HIV coinfection, T_REG_ accumulate in the intestines during CMV colitis [142], but it is unclear whether these cells influence pathogenesis. Data from the MCMV model suggest that T_REG_ cells may impact mucosal CMV chronicity. Conditional depletion of T_REG_ cells during persistence in SGs increases T-cell activation and associated control of virus replication [143]. Interestingly, T_REG_ cells may play different roles in different CMV-infected tissues during viral latency. T_REG_ depletion during MCMV infection latency revealed that T_REG_ cells limit CMV reactivation and subsequent replication in SGs, whereas in the spleen, T_REG_ antagonize CD8^+^ T-cell effector function and promote virus carriage [144].

### Paradoxical functions of T_REG_ in HIV infection

The role of T_REG_ cells in HIV-1 infection has been extensively investigated, with the hypothesis that these cells, through their durability and ability to suppress cell-mediated immunity, may represent a major reservoir for HIV [145]. Uncontrolled HIV-1 replication is associated with increased numbers of GALT T_REG_ cells [146], and nonhuman primate studies suggest that these cells contribute to SIV persistence [147]. While there is evidence to support the latent infection of T_REG_ cells in peripheral blood [148], HIV latency in mucosal T_REG_ cells has not been established. Elite controllers and long-term nonprogressors have lower levels of T_REG_ cells in mucosal tissues [149, 150], suggesting that T_REG_ may suppress antiviral T-cell responses in HIV infection.

These data imply a classical anti-inflammatory role for T_REG_ in HIV infection. Paradoxically, most studies in patients with uncontrolled HIV indicate a positive relationship between T_REG_ frequencies and immune activation [151]. Whether the latter findings reflect a futile inhibitory response in the face of overwhelming immune activation is unclear. However, functionally suppressive T_REG_ cells are elevated in pediatric slow progressors, who maintain high CD4^+^ T-cell counts and low immune activation despite high viral loads, suggesting a role for T_REG_ cells in preventing disease progression [152]. In the endocervix, T_REG_ cells are associated with decreased genital inflammation and a decreased abundance of CD4^+^ T cells, which has been speculated to lower the risk of HIV acquisition [153]. Thus, it is possible that, akin to CMV, T_REG_ cells during HIV infection may exhibit differing functions or at least exert differing effects on the antiviral immune response at mucosal and nonmucosal sites.

The complexity of T_REG_ biology has been further demonstrated in chronic oncogenic viral infections. Human papillomavirus (HPV) is a common sexually transmitted viral infection causing anogenital warts that, in a subset of individuals who acquire high-risk serotypes, may establish latency in epithelial cells and can result in neoplasia [154]. T_REG_ cells accumulate more frequently in larger warts, and depletion of FoxP3^+^ T cells enhances the responsiveness of infiltrating effector T cells [155]. Likewise, T_REG_ cells accumulate in cervical cancer, the majority of which are induced by high-risk HPV serotypes, with particularly high frequencies seen in patients with lymph node metastases [156, 157]. Supporting the putative role of T_REG_ cells in promoting carcinogenesis in latent HPV infection, FoxP3 expression increases during progression of precancerous cervical intraepithelial neoplasia to invasive squamous cell carcinoma [158] and is associated with lymphangiogenesis [159]. The mechanism linking T_REG_ cells and HPV-driven carcinogenesis is unclear. In HPV-associated squamous cell carcinoma of the head and neck, T_REG_ cells have been observed to have both positive and negative prognostic associations depending on the anatomical site (reviewed previously [160]), supporting the notion that the local and tissue-specific immune context can skew T_REG_ cells to more predominantly suppress either effector or regulatory immune cells.

Finally, the nuanced role that T_REG_ cells play in mucosal herpesvirus infection has been highlighted in murine studies of HSV-2. T_REG_ cells are required for the trafficking of dendritic cells from the vaginal mucosa to draining lymph nodes, with T_REG_ depletion leading to impaired CD4^+^ T-cell priming [161]. Consistent with the function of T_REG_ in orchestrating antiviral mucosal T-cell immunity, Tr1 cells expressing T-bet have been shown to promote CD8^+^ T_RM_ development during pulmonary influenza infection [162]. Thus, in certain contexts, T_REG_ cells may orchestrate and not inhibit mucosal antiviral T-cell immunity.

## Mucosal-associated invariant T cells

MAIT cells are a population of innate-like T cells restricted by the highly conserved MHC class I-related (MR1) molecule and express a semi-invariant T-cell receptor α-chain (Vα7.2–Jα33/20/12 in humans). They are most abundant in the blood and liver, but they are enriched in mucosal surfaces, and discordant TCR-β chain expression between these compartments implies functional heterogeneity [163]. The main ligands presented by MR1 to MAIT cells are derived from microbial riboflavin biosynthesis [164], so viruses, that do not generate these ligands, cannot be recognized by the MAIT cell TCR. However, TCR-independent MAIT cell activation can be triggered by inflammatory cytokines [165] and engagement of Toll-like receptors [166]. MAIT cells have garnered much interest, and their role in chronic viral infection is becoming increasingly recognized [167]. Peripheral MAIT cells are persistently depleted in chronic HIV-1 infection [168] and exhibit abnormal T-Bet and Eomes expression associated with impaired cytotoxic and proliferative capacity [169]. In contrast, MAIT cells in the gastrointestinal tract may be relatively preserved and/or restored by ART [170, 171], but their role in mediating protection is unknown. Further study of MAIT biology may reveal a novel immunotherapeutic option in infections where MAIT cells are impaired. In the context of HIV-1, this may be complicated by the fact that MAIT cells may serve as a viral reservoir [172]. MAIT cells have also been studied in the context of other viral infections (reviewed elsewhere [167]), and HCMV can downregulate MR1 expression through a number of mechanisms, including expression of the viral glycoprotein gpUS9 [173].

## Invariant natural killer T cells

Invariant natural killer T (iNKT) cells are innate-like T cells that express an invariant T-cell receptor restricted by the MHC class I-related protein CD1d and that present microbial-derived glycolipid antigens, including α-galactosylceramide (α-GalCer). As with MAIT cells, viruses do not produce these antigens and thus are not thought to directly engage the iNKT TCR. However, iNKT cells may be activated in a TCR-independent manner by virally induced cytokines [174]. In HIV-1-infected individuals, there is preferential loss of anti-inflammatory CD4^+^ iNKT cells in the colonic mucosa correlated with systemic immune activation [175], and these cells are not restored to normal after ART initiation [176]. In chronic HIV infection, iNKT cells may become exhausted, with upregulation of PD-1 and LAG-3 and irreversible loss of functionality even after ART treatment [177]. Immune checkpoint blockade reverses this phenotype in vitro [178]. iNKT cells have also been studied in the context of MCMV infection, and evidence shows that adjuvant administration of α-GalCer at the time of infection reduces viral titers and promotes CD8^+^ T_CM_ accumulation, although these authors studied the spleen and liver and not mucosal tissues [179].

Further study is needed to fully elucidate the contributions of these unconventional T cells to the control of chronic viral infections, particularly those of the mucosa. However, the fact that viruses, including HSV-1, HCMV and HIV-1, employ immune evasion mechanisms to downregulate MR1 and/or CD1d expression [173, 180–183] is suggestive of the evolutionary importance of these cells in countering these chronic viral infections.

## γδ T cells

γδ T cells are a diverse group of unconventional T cells that have evolved to recognize a variety of (only partially characterized) molecules derived from stressed, transformed or infected cells [184]. These include non-MHC-restricted viral antigens, for example, glycoprotein I produced by HSV-1 [185]. This T-cell subset is developmentally programmed to home to peripheral tissues, including mucosal tracts [186]. The role played by γδ T cells in mediating control of mucosal immunity more broadly has been extensively reviewed elsewhere [187].

In the context of chronic viral infections, γδ T cells have been extensively studied in solid-organ transplant recipients who develop CMV disease. This response is complex, with different γδ T-cell subtypes mediating heterogeneous roles. For example, Vδ2^−^ γδ T cells, which are predominantly located in the intestinal and skin epithelia, are able to recognize CMV-infected cells, exhibit cytotoxic activity and undergo considerable expansion [188], and are correlated with resolution of infection [189]. Similarly, peripheral Vγ9^-^Vδ2^+^ T cells have recently been shown to respond to CMV-infected cells, but unlike Vδ2^-^ γδ T cells, their frequency appears to correlate with CMV disease severity [190]. Viral infection results in clonal expansion and effector differentiation of Vγ9^−^Vδ2^+^ T cells, identifying these cells as adaptive-like [191]. In contrast, Vγ9^+^Vδ2^+^ T cells do not respond to CMV-infected cells, and through their semi-invariant TCR repertoire and public Vγ9 TCR sequence, these cells have been described as innate-like [191].

How γδ T cells respond to and contribute to antiviral protection against CMV infection is incompletely understood. CMV infection in utero induces the expansion of peripheral fetal γδ T cells, which express high levels of IFNγ, the transcription factors T-Bet and EOMES, natural killer receptors, and cytotoxic mediators [192]. Additionally, a specific Vδ2^−^ clone (Vγ4Vδ5) can recognize CMV-infected (and transformed) cells through direct binding of the MHC-like endothelial protein C receptor (EPCR) in the absence of an EPCR-presented ligand. While this subset of Vδ2^−^ T cells may be enriched in some mucosal tissues, such as the lung [193] and gut [194], the extent to which they contribute to mucosal immunity in CMV disease is unclear. Adding further complexity, the EPCR findings were derived from studies of an HCMV strain containing deletions in genes encoding several viral proteins, including UL148 and UL148D. These genes are present in wild-type HCMV and were recently shown to target the protease A disintegrin and metalloproteinase 17 (ADAM17) and to regulate the expression of more than 100 cell surface proteins, including EPCR [195]. As such, Vγ4Vδ5 T-cell-EPCR binding may not be relevant for HCMV infection.

The adaptive nature of the antiviral γδ T-cell immune response is evidenced by the clonal proliferation of peripheral CMV-reactive γδ T-cells in patients following reconstitution in hematopoietic stem cell transplant recipients who developed CMV infection [196]. Furthermore, recent evidence in kidney transplant recipients suggests that the relative protection from CMV infection conferred by mTOR inhibitors may be partly explained through the enhancement of peripheral γδ T-cell cytotoxicity [197]. Further work is needed to determine the precise functional roles of these cells and whether similar reconstitution occurs in mucosal tissues. Experimental evidence for an antiviral function for these responses in CMV infection has been reported using the MCMV model, in which adoptive transfer of γδ T cells from MCMV-infected mice confers lasting protection following CMV infection in RAG-1 knockout mice, which is associated with the accumulation of these cells in tissue sites, including the lungs and intestines [198, 199]. The mechanism of this protection was not clear, but it appeared to be independent of NK, B and αβ T cells.

The extent to which γδ T cells contribute to the control of viraemia and/or disease progression in HIV-1 infection is unclear. Among controllers, Vδ1 γδ T cells can be identified in the gut mucosa; these cells predominantly exhibit an effector memory phenotype and produce cytokines, including IFNγ, TNF, and MIP-1β [200]. In vitro, γδ T cells inhibit HIV-1 replication and spread [201]. Among gut-associated γδ T cells, chronic HIV infection is associated with decreased functionality, as measured by CD107a and IFN-γ expression, compared to acute infection, suggesting that these cells assume the exhausted phenotype seen in other T-cell subsets [202]. There is conflicting evidence regarding the impact of HIV infection on mucosal γδ T‐cell population size, with reports of increases in Vδ1 T cells and decreases in Vδ2 T-cell frequency in the rectal mucosa [203] contrasting with an apparent decrease in Vδ1 cells in duodenal samples [202]. Both Vδ1 and Vδ2 populations are reduced in the endocervical mucosa of predominantly ART-treated women [204]. The functional implications of changes in mucosal γδ T‐cell frequencies are not known.

## Exploiting chronic virus infections in the development of T-cell-inducing vaccines for infectious disease

The detrimental impacts of chronic virus infections in mucosal tissues in terms of dissemination and pathogenesis are clear. The features of mucosal T-cell responses induced by certain chronic viral infections can also be harnessed in vaccination strategies to protect mucosal surfaces from other infectious diseases, including those discussed herein and others, such as SARS-CoV2 [205]. Therefore, viruses that persist in the mucosa and/or induce robust and long-lived T-cell responses at these sites could be exploited in vaccine development.

## Cytomegalovirus

The ability of CMV to induce robust and long-lived functional memory T-cell responses has led to interest in developing CMV-based vaccines against infectious agents and tumors (reviewed elsewhere [206, 207]). Importantly, the ability of CMV to induce high frequencies of antigen-specific T cells, including T_RM_, on mucosal surfaces that are maintained over time differentiates this approach from established vaccine platforms that have come to the fore during the COVID-19 pandemic, as the latter induce T-cell responses that wane over time.

### CMV-based vaccine vectors elicit protection from heterologous mucosal virus challenge

The potential utility of CMV as a vaccine vector was first demonstrated in the SIV model of HIV pathogenesis. A key goal for any HIV vaccine will be the prevention or rapid control of virus replication at the mucosal portal of entry. One approach to this problem, given the contribution of mucosal CD8^+^ T cells to HIV infection, is to elicit strong HIV-specific T-cell responses in the mucosa and beyond. The intrarectal SIV infection model has been used to study this problem using RhCMV vectors engineered to express SIV antigens (Gag, Rev-Tat-Nef and Env). These vectors could superinfect RhCMV immune hosts and induce large frequencies of SIV-specific CD4^+^ and CD8^+^ T_EM_ cells [208]. Excitingly, vaccinated macaques showed resistance to SIV infection, and a later study using highly pathogenic intrarectal SIV challenge demonstrated that ~50% of vaccinated macaques completely controlled SIV infection [209]. Despite the known induction of high frequencies of canonical CD8^+^ T cells by CMV, later studies by the Picker group revealed that SIV-specific CD8^+^ T cells induced by their RhCMV vector recognized only MHC class II and HLA-E restricted epitopes and not peptides presented by classical HLA molecules [210]. Indeed, abrogation of the priming of HLA-E-restricted CD8^+^ T cells resulted in loss of vaccine efficacy, suggesting that these responses mediate protection in this model [211].

Evidence regarding the relative contribution of these unusual T-cell responses in the mucosa specifically came from a detailed analysis of macaques that ultimately controlled SIV infection after intrarectal dissemination. In these primates, virus spread beyond the mucosal port of entry, with virus detected in plasma, mucosal-draining lymph nodes, bone marrow, spleen and/or liver [209]. Thus, the presence of RhCMV-induced mucosal T cells was insufficient to completely control initial SIV replication at the mucosal port of entry. The authors also examined intravaginal SIV challenge where virus amplification in the mucosa is required prior to systemic virus dissemination after 4–5 days [212]. Here, again, prior vaccination with the RhCMV-based construct induced robust SIV-specific CD4^+^ and CD8^+^ T-cell responses, as seen in previous studies; little or no SIV-specific antibody production; and some vaccinated macaques controlled SIV infection after challenge. As with intrarectal challenge, ~50% of these ‘controllers’ of intravaginal SIV infection exhibited virus spread beyond the mucosa. Overall, these data suggest that mucosal *and* systemic vaccine responses contribute to HIV control.

One important consideration is that these RhCMV-based vaccines were administered via the subcutaneous route. As a general principle, one may predict that mucosal T-cell immunity induced by CMV-based vaccine vectors may be enhanced by mucosal administration. In the context of using CMV-based vaccines to induce heterologous respiratory viral infections, this question has been directly addressed using the MCMV model by the Graham and Čičin-Šain groups, who developed MCMV-based vaccines expressing antigens from respiratory syncytial virus (RSV) [213] and Influenza [214], respectively. In both systems, mucosal but not systemic administration of MCMV-based vaccines was required for protection from heterologous viral challenge, and this protection was associated with increased induction of CD8^+^ T_RM_ cells within the lung. Experiments using the local depletion of pulmonary T cells [214] or blockade of T-cell egress from lymph nodes using a sphingosine-1-phospate receptor 1 agonist [213] provided evidence for the requirement of CMV-induced T_RM_ cells for protection. Notably, however, the requirement for CMV-induced T_RM_ cells in vaccine-induced antiviral immunity against heterologous respiratory viral infections is dependent on the virus against which immunity is elicited. Oxenius and colleagues demonstrated that protection from vaccinia virus could be achieved following systemic vaccination with an MCMV-based vector that induced T_EM_ responses [215]. Vaccine-induced T cells localize close to the vasculature and, upon vaccinia challenge, extravasate into the lung parenchyma in a manner partly dependent upon CXCR3 [215]. Thus, in certain contexts, CMV-induced T_EM_ cells appear sufficient for eliciting protection against heterologous viral challenge.

### Immunogenicity of attenuated CMV vectors

The importance of CMV vaccine-induced T_RM_ cells in protection from some heterologous mucosal infections and the requirement for chronic antigenic stimulation for the induction and maintenance of T_RM_ cells suggest that chronic CMV replication is a fundamental requirement for the protective efficacy of CMV-based vaccines. However, HCMV pathogenesis remains a major concern for the development of such approaches in humans, particularly given that pathogenesis in mucosal organs represents a disease manifestation of CMV in certain individuals [35]. Thus, nonreplicating CMV vectors may be required for clinical translation. This approach was first explored in an MCMV model, where a spread-deficient virus lacking the essential gene encoding M94 induced robust CD4^+^ and CD8^+^ T-cell responses specific for exogenous (OVA) protein following systemic vaccination [216]. Moreover, MCMV lacking the viral glycoprotein L (gL), which is required for cell-to-cell spread, induces memory T-cell responses following infection via the intraperitoneal [217] or subcutaneous route [218] of administration, albeit to a lesser degree than the responses induced by replicating viruses.

In RhCMV, a vector lacking the tegument protein pp71, which results in an ~1000-fold reduction in virus replication in vivo, induced comparable CD8^+^ T-cell responses and only marginally reduced CD4^+^ T-cell responses, including those in the respiratory mucosa [219]. Subcutaneous vaccination with this vector afforded comparable protection from intravaginal SIV challenge to that afforded by immunization with a replicating vector, including following multiple challenges over a 3-year period [220]. While this was encouraging, some virus replication was still detectable. To negate this risk, a RhCMV vector was generated that only replicates in the presence of the FK506 analog Shield-1. Although this vector induced durable T_EM_ CD8^+^ T-cell responses restricted by classical MHC, this replication-deficient vector did not elicit HLA-E-restricted T-cell responses and subsequently failed to protect against SIV challenge [221].

Similarly, Merck Sharp & Dohme (MSD) developed a replication-defective HCMV construct (V160) based on the Shield-1 system for vaccination from congenital HCMV infection [222]. V160 induced virus-specific neutralizing antibody and T-cell responses following intramuscular or intradermal vaccination [223]. Disappointingly, however, recent data from a phase 2b clinical trial reported that, compared with a placebo, V160 did not reduce the incidence of congenital CMV infection [224]. The reasons for these results are not clear. Data from a phase 1 study demonstrated that peripheral IFNγ T-cell responses induced by V160 were comparable to those induced by natural infection, although the relative contributions of CD4^+^ and CD8^+^ T-cell responses to this result was unclear, and the induction of nonclassical T cells and mucosal T-cell responses (and inhibitory T cells) was not measured [223].

**Fig. 3:**
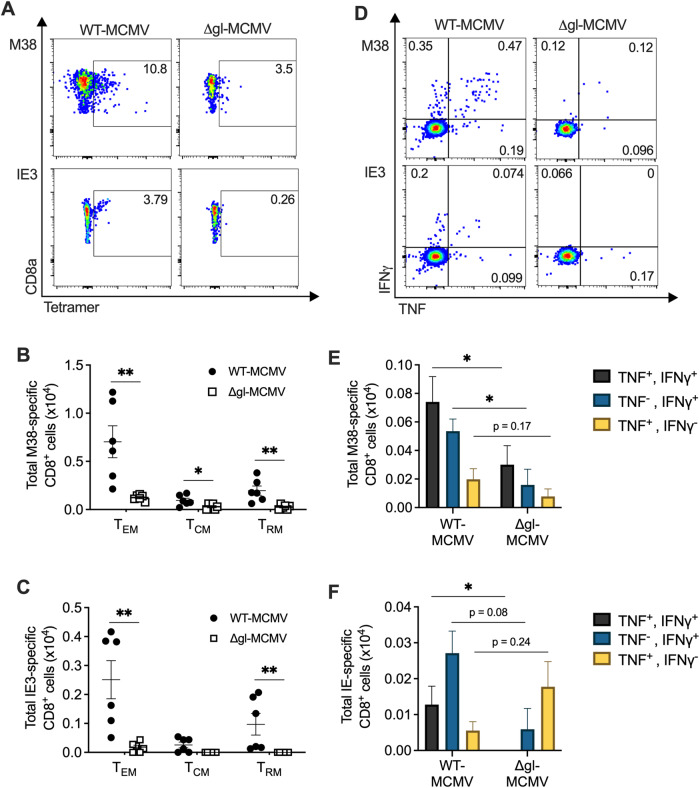
Cytomegalovirus (CMV) replication is required for optimal induction of CD8^+^ T-cell memory formation in the lungs following mucosal infection. C57BL/6 mice were intranasally infected with 2 × 105 PFU of WT-MCMV or ΔgL‑MCMV. Leukocytes were isolated from perfused lungs 9 weeks post infection, and T-cell responses were quantified using flow cytometry. Representative concatenated FACS plots of tetramer-bound CD8^+^ T cells reactive to M38 or IE3 (**A**). Numbers of tetramer-binding effector memory T cells (TEM: CD8^+^ CD3^+^ CD44^+^ CD62L^−^), central memory T cells (TCM: CD8^+^ CD3^+^ CD44^+^ CD62L^+^) and tissue resident memory T cells (CD8^+^, CD3^+^ CD69^+^, CD103^+^) reactive to M38 (**B**) or IE3 (**C**). Data shown as mean +/− SEM (*n* = 6 mice per group). *p ≤ 0.05, **p ≤ 0.01, Mann‒Whitney unpaired *t* test. **D** Concatenated flow cytometry plots of IFNγ and TNF-α expression by M38- and IE3-specific CD8^+^ T cells in the lungs of mice infected with WT-MCMV or ΔgL‑MCMV. Total numbers of polyfunctional and monofunctional T cells in the lungs gated on live CD8^+^ T cells reactive to M38 (**E**) or IE3 (**F**). *p ≤ 0.05, **p ≤ 0.01, ordinary one-way ANOVA for multiple comparisons. All data represent 2–3 independent experiments

All of these data were derived from systemic vaccinations. Given the possible requirement for mucosal administration of CMV-based vaccines for protection from certain infections, it is also important to understand whether effective induction within the mucosa following mucosal vaccination can be achieved without virus replication. To address this, we infected C57BL/6 mice with ΔgL-MCMV or wild-type (WT) MCMV intranasally. After 9 weeks, we assessed the induction of virus-specific CD8^+^ T-cell responses in the lungs, focusing on the inflationary MCMV-specific CD8^+^ T-cell responses to H2Kb-restricted MHC class I-restricted peptides derived from M38 and IE3. Tetramer staining of leukocytes derived from perfused lungs revealed a significant reduction in the frequency of M38- and IE3-specific CD8^+^ T cells in ΔgL-MCMV-infected mice compared to WT-MCMV-infected mice (Fig. 3A). As expected [115], the majority of cells exhibited a T_EM_ phenotype, with some T_RM_ cells and a few central memory T cells (T_CM_) also present (Fig. 3B, C). In accordance with the overall reductions in the frequencies and total numbers of CD8^+^ T cells specific for both viral epitopes, virus-specific T_EM_, T_CM_ and T_RM_ cells were significantly rarer in the ΔgL-MCMV-infected mice (Fig. 3B, C). Thus, these data suggest that active virus replication and spread are required for optimal local generation of T_EM_ and T_RM_ CD8^+^ T cells.

In addition to T-cell accumulation, virus replication in the mucosa was required for optimal cytokine production by virus-specific T cells. Specifically, the proportion and total number of both M38- and IE3-specific CD8^+^ T cells in the lungs that coexpressed IFNγ and TNF were reduced by the absence of virus replication (Fig. 3D–F). Taken together, these data indicate that viral replication is required for the accumulation of functional memory T cells in the lungs following mucosal MCMV challenge/immunization.

### Possible strategies to enhance the mucosal immunogenicity of replication-deficient CMV vectors

**Fig. 4:**
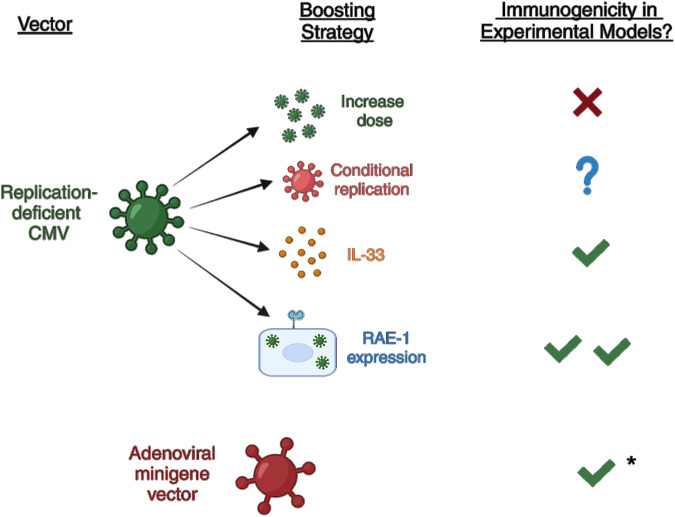
Possible approaches to enhancing the mucosal immunogenicity of replication-deficient CMV vectors. A schematic illustrating the approaches used to increase the immunogenicity of spread-deficient CMV vectors. All data were derived from nonmucosal vaccination routes. *Adenoviral minigene vectors induce inflationary CD8^+^ T-cell responses, but side-by-side comparisons with replication-deficient CMV vectors have not been performed

Overall, the data derived from replication-deficient vectors in different clinical and experimental settings and from different vaccination routes suggest that these vectors may afford insufficient protection from mucosal viral challenge. There are several possible solutions to enhance the immunogenicity of these vectors (Fig. 4). The first, simply, is to administer a greater dose of the replication-deficient vector. However, this approach has minimal impact on improving the immunogenicity of subcutaneous ΔgL-MCMV immunization [218]. Using inducible replication systems such as Shield-1 to induce controlled vector replication represents an alternative strategy to increasing vaccine immunogenicity. Alternatively, IL-33 is a member of the IL-1 family of cytokines that acts as an alarmin when released in response to infection or cell stress [225] and promotes the induction of antiviral CD8^+^ T-cell immunity [226]. We identified that IL-33 expression is induced in secondary lymphoid tissues in response to systemic MCMV infection [227]. Importantly, in this study, MCMV replication was a key requirement for IL-33 induction. Administering recombinant IL-33 as an adjuvant increased the induction of effector CD8^+^ memory T-cell responses after systemic ΔgL-MCMV administration, and this adjuvant approach improved protective immunity in response to a heterologous systemic virus challenge [227]. Interestingly, in accordance with the known role of IL-33 in the induction of CD69 [228], IL-33 administration also increased the accumulation of virus-specific (CD103^+^ CD69^+^) CD8^+^ T_RM_ [227], suggesting that the use of IL-33 could be explored to circumvent the requirement for virus replication in the mucosa in CMV-based vaccination strategies.

Another approach to inducing mucosal T-cell responses without the risk of chronicity of the viral vector is to engineer vectors to express immune-stimulatory ligands. Jonjić and colleagues demonstrated that engineering MCMV to express RAE-1γ, a ligand for the NK cell receptor NKG2D, in place of the viral gene m152, which would otherwise downregulate RAE-1γ and thus inhibit NKG2D activation, significantly attenuates MCMV [229]. Interestingly, despite attenuated virus replication, RAE-1γ-expressing MCMV actually exhibited improved immunogenicity and T-cell-mediated protection against *Listeria monocytogenes* challenge [230], implying that replication-deficient CMV vectors can be engineered to induce comparable, or perhaps superior, responses to those induced by replicating vectors.

## Adenovirus

Robust mucosal T-cell responses characteristic of CMV-induced responses may also be induced by other viral vectors with more favorable safety profiles. One vaccine platform to come to the fore in the COVID-19 pandemic was adenovirus. Human adenovirus (Ad) is an acute infection that usually causes mild respiratory illness, although it is known to cause more severe respiratory illness and skin and gastrointestinal infections, particularly in immunosuppressed individuals [86]. Three human adenovirus-based vaccines derived from Ad26 (Ad26.COV2. S), Ad5/Ad26 (Gam-COVID-Vac), Ad5 (Ad5-nCOV) and chimpanzee adenovirus Y25 (ChAdOx1 nCoV-19) were developed in the SARS-CoV-2 pandemic, all of which exhibited significant clinical efficacy in association with the induction of both T-cell and humoral anti-SARS-CoV-2 immunity [231].

Despite the fact that Ad causes acute infection, viral antigen can persist for weeks after intramuscular and intravenous immunization with Ad vectors (reviewed elsewhere [232]). Experiments studying Ad5 immunogenicity in mice after intravenous infection demonstrated that vectors engineered to express a single MHC class I-restricted epitope induced inflationary epitope-specific T-cell responses that exhibited T_EM_-like features comparable to those observed in CMV infection [233, 234]. As seen in MCMV [68], persistent expression of antigen appears essential for Ad5-induced inflationary T-cell responses [235]. Similarly, inflationary CD8^+^ T-cell responses induced by Ad are IL-33 dependent, and pulmonary fibroblasts are an important source of this cytokine [236]. Thus, although adenovirus infection is considered an acute viral infection, vaccine vectors based on this virus express antigens persistently within mucosal tissue and thus potentially safely induce robust mucosal CD8^+^ T-cell responses. Excitingly, intranasal administration of ChAdOx1-S expressing the full-length SARS-CoV-2 Spike protein in BALB/c mice elicited a superior mucosal immune response compared to intramuscular vaccination, as measured by greater serum and mucosal IgA titers, proportion of epitope-specific resident CD8^+^ T cells and ability of bronchoalveolar lavage fluid from these mice to neutralize SARS-CoV-2 in vitro [237]. It will be interesting to examine whether these Ad-based approaches induce robust mucosal T-cell responses against other viral challenges when administered via mucosal routes.

## Considerations of the utility of mucosal T-cell-inducing virus-based vaccines

A vaccinated individual will be exposed to and need to mount immune responses to a multitude of infectious challenges during their life course. As such, an important consideration for all T-cell-inducing vaccines designed to protect mucosal surfaces from infections, irrespective of attenuation, is the question of how dominant a T-cell response to a single pathogen should be. This is of particular relevance given the association between inflationary T-cell responses against CMV and immunosenescence [88]. The argument for inducing very strong T-cell responses to HIV is perhaps justified, particularly given the detrimental effect that HIV has on heterologous immune responses to other pathogens. Furthermore, although not discussed herein, viral-based vaccine vectors are also being explored in cancer, and the strategy of inducing high-frequency T-cell responses to various cancers, including those in mucosal surfaces, also appears reasonable, particularly in the context of therapeutic vaccination or prophylactic strategies following initial tumor resection. In the case of respiratory viral infections such as SARS-CoV-2 or influenza, how large a T-cell response would one actually want to induce? As highlighted by studies using MCMV vectors, understanding the relative contribution of T_EM_ and T_RM_ cells to antiviral protection from the pathogen that one wishes to induce protection against will be important for defining the optimal vaccination strategy. Understanding how large a T-cell response is required for protection will also guide vector choice, including dose, attenuation strategy and possible route of immunization.

## Conclusions

T cells within mucosal tissues clearly influence the pathogenesis of numerous chronic viral infections. The evolution of mucosal T-cell responses, such as through the induction of regulatory T-cell populations or the development of T-cell exhaustion, typically impinges on antiviral immunity but may represent an intricate balance to limit immunopathology. Ultimately, mucosal T cells are critical front-line defenders against numerous viruses that target mucosal surfaces to establish infection, and successful vaccines that target such viruses will need to induce these responses. Experimental evidence suggests that the relative contributions of T_RM_ and T_EM_ cells to protective immunity varies between pathogens and possibly mucosal sites. Although murine and other animal models have clear limitations, particularly in terms of studying nonclassical T-cell biology, using experimental models in combination with clinical data will help determine the optimal mucosal T-cell response to be induced by vaccination and will inform the types of vaccines and the routes of administration required to induce effective responses against pathogens of global clinical significance.
